# No evidence of involvement of E-cadherin in cell fate specification or the segregation of Epi and PrE in mouse blastocysts

**DOI:** 10.1371/journal.pone.0212109

**Published:** 2019-02-08

**Authors:** Katarzyna Filimonow, Nestor Saiz, Aneta Suwińska, Tomasz Wyszomirski, Joanna B. Grabarek, Elisabetta Ferretti, Anna Piliszek, Berenika Plusa, Marek Maleszewski

**Affiliations:** 1 Department of Embryology, Faculty of Biology, The University of Warsaw, I. Miecznikowa, Warsaw, Poland; 2 Division of Developmental Biology and Medicine, The University of Manchester, Oxford Road, Manchester, United Kingdom; 3 Department of Experimental Embryology, Institute of Genetics and Animal Breeding, Polish Academy of Sciences, Postępu 36a, Jastrzębiec, Poland; 4 Faculty of Biology, Biological and Chemical Research Centre, The University of Warsaw, Zwirki i Wigury, Warsaw, Poland; 5 The Danish Stem Cell Centre (DanStem), University of Copenhagen, Blegdamsvej, Copenhagen N, Denmark; University of Otago, NEW ZEALAND

## Abstract

During preimplantation mouse development stages, emerging pluripotent epiblast (Epi) and extraembryonic primitive endoderm (PrE) cells are first distributed in the blastocyst in a “salt-and-pepper” manner before they segregate into separate layers. As a result of segregation, PrE cells become localised on the surface of the inner cell mass (ICM), and the Epi is enclosed by the PrE on one side and by the trophectoderm on the other. During later development, a subpopulation of PrE cells migrates away from the ICM and forms the parietal endoderm (PE), while cells remaining in contact with the Epi form the visceral endoderm (VE). Here, we asked: what are the mechanisms mediating Epi and PrE cell segregation and the subsequent VE *vs* PE specification? Differences in cell adhesion have been proposed; however, we demonstrate that the levels of plasma membrane-bound E-cadherin (CDH1, cadherin 1) in Epi and PrE cells only differ after the segregation of these lineages within the ICM. Moreover, manipulating E-cadherin levels did not affect lineage specification or segregation, thus failing to confirm its role during these processes. Rather, we report changes in E-cadherin localisation during later PrE-to-PE transition which are accompanied by the presence of Vimentin and Twist, supporting the hypothesis that an epithelial-to-mesenchymal transition process occurs in the mouse peri-implantation blastocyst.

## Introduction

The formation of extraembryonic lineages that facilitate the establishment of mother-foetus connections and participate in the interchange of nutrients and metabolites within the maternal uterine environment is a prerequisite for the successful development of mammalian embryos [1]. The first extraembryonic epithelium, the trophectoderm (TE), has been extensively studied in recent years [2]; however, our knowledge of the mechanisms leading to the formation of the second extraembryonic lineage, the primitive endoderm (PrE), remains limited.

PrE precursors differentiate within the inner cell mass (ICM) of mammalian blastocysts before implantation. Simultaneously to that differentiation, the remaining ICM cells specify the embryonic epiblast (Epi) lineage, which will give rise to the body of the future foetus after implantation [3,4]. Specification of the PrE and Epi lineages in the mouse embryo is a multi-step process. At the early blastocyst stage (~32 cells) PrE- and Epi-specific genes (*Gata6* and *Nanog*, respectively) are co-expressed in all ICM cells [5,6]. Starting from around the 64-cell mid-blastocyst stage, downregulation of the genes involved in pluripotency in PrE precursors and downregulation of *Gata6* in Epi precursors initiate appropriate cell fate specification and the emergence of precursors of both lineages, which are initially randomly distributed throughout the ICM [5–8]. It has recently been shown that individual precursor commit to the Epi or PrE lineages asynchronously [9]. In the late blastocyst stage (>100 cells), PrE and Epi cells become segregated into separate compartments: PrE cells form an epithelial monolayer on the surface of the ICM facing the blastocyst cavity, whilst Epi cells remain encapsulated by the PrE and overlying polar TE cells [6, 10, 11]. After implantation, a subset of PrE cells migrates from the ICM to give rise the parietal endoderm (PE) [12], while the remaining PrE cells at the surface of the ICM form the visceral endoderm (VE) [13, 14]. Subsequently, PE cells secrete basement membrane proteins to form Reichert’s membrane [15, 16]. The VE, in turn, partially develops into the endodermal membrane of the visceral yolk sac [17] and assists in gas and nutrient exchange between the growing embryo and its environment, as well as in patterning of the embryo [1].

It has been suggested that PrE-to-PE transition involves PrE cells undergoing a process of epithelial-to-mesenchymal transition (EMT) [18–20]. However, experimental proof that this process indeed takes place during PrE-to-PE is lacking. EMT is a multi-step cell-remodelling process that occurs during organogenesis and numerous pathological processes, such as cancer metastasis. During EMT, polarised epithelial cells lose their epithelial properties and acquire the migratory capabilities typical of mesenchymal cells [21]. The first step requires that cells lose the cell junctions typical of epithelia, as well as downregulate intra-cellular apical-basal polarity [22]. Indeed, the loss of E-cadherin protein from the plasma membrane, coupled with the inactivation of *E-cadherin* gene transcription by factors like Snail or Twist, are two recognised hallmarks of EMT events [21–23]. Moreover, downregulation of *E-cadherin* is usually followed by the characteristic switch towards the expression of other cadherins, such as *N-cadherin* [21]. Subsequently, transitioning cells acquire migratory properties, since the cytoskeleton of the cells is reorganised. Actin filaments are remodelled, and Vimentin intermediate filaments replace cytokeratin intermediate filaments, which allows cells to detach and move away from their original host tissue sites. After successfully reaching their appropriate niches, the cells transform back into an epithelium, a process which is essentially the opposite of EMT and which is referred to as mesenchymal-epithelial transition (MET) [21].

E-cadherin, a member of a superfamily of calcium-dependent transmembrane proteins, plays a pivotal role in regulating cellular adhesion, as it is required for the formation of adherens junctions [24]. In mice, E-cadherin is required during embryo compaction and the maturation of the TE [25–28]. E-cadherin is also associated with the actomyosin cortex [29–31] and is a mediator of periodic cortical waves, which act as the primary source of the compactive forces [32]. Changes in E-cadherin localisation during compaction are also necessary for the acquisition of intra-blastomere polarity, as reflected by the apical distribution of proteins such as PAR3, PAR6, Ezrin, aPKC and Actin [25, 27, 33–37]. These changes are absolutely required for TE specification [27]. Recently, higher expression levels of actomyosin complex components have been linked to elevated levels of blastomere contractility, which in turn ultimately causes cells to contribute to the ICM [38]. Although the role of E-cadherin during TE formation is well known [27], its function in the differentiation and segregation of Epi and PrE cells has not been studied.

It has been proposed, but never unequivocally reported, that differential cell adhesion is important for the segregation of Epi and PrE cells [6–8, 39–41]. As E-cadherin is a major adhesion protein in mammalian embryos, we investigated its distribution between PrE and Epi cells within the mouse blastocyst ICM. First, we report that both Epi and PrE precursor cells did not differ in terms of E-cadherin expression and localisation. However, once PrE and Epi cells were fully segregated to their separate tissue layers, we observed lower levels of membrane E-cadherin in neighbouring cells of the PrE compartment than in those of the Epi compartment. Additionally, we analysed the consequences of *E-cadherin* downregulation during active segregation of PrE and Epi cells and discovered that such cell adhesion interference did not affect the segregation of these two ICM cells populations. Finally, we hypothesized that E-cadherin may be involved in the later PrE-to-PE *vs* PrE-to-VE transition. The observed pattern of E-cadherin localisation in PE cells was indeed consistent with cells undergoing an EMT process [21]. Moreover, the distinct lack of E-cadherin coupled with the presence of other EMT markers, such as Vimentin and Twist in PrE cells, supports the hypothesis that an EMT process may contribute to lineage specification events (*i*.*e*., PrE-to-PE transition) during the peri-implantation stage of mouse embryo development.

## Materials and methods

### Embryo collection and culture

The following mice strains were used: F1 (C57Bl/6xCBA/H) (University of Warsaw), CD1 and transgenic Pdgfrα^H2B-GFP/+^ [42] or CAG:GPI-GFP [43] (University of Manchester). In both Animal Facility Units mice were kept under a 12 hour light cycle and were given access to food and water *ad libitum*. 8- to 12-week-old female mice were mated with males from the same strain. The presence of the vaginal plug was used to indicate pregnancy, defining the first day as embryonic day 0.5 (E0.5). Embryos from natural mating were recovered in homemade M2 medium [44]. Pregnant females were sacrificed by cervical dislocation at the desired stage of embryonic development. Oviducts and uterine horns were collected into sterilised culture dishes. 8-cell stage embryos were collected from oviducts by flushing, whereas other stages were collected from the uterus by flushing (E3.5–E4.75) or microdissection (E5.5). For immunostaining, embryos were recovered at the following stages: E3.5, E3.75, E4.25, E4.5, E4.75 and E5.5. For RNA injections, 8-cell stage embryos were recovered at E2.25. All embryos were cultured in KSOM-AA (Millipore) [45, 46] drops under mineral oil at 37.5°C and in an atmosphere supplemented with 5% CO_2_.

All mouse handling and husbandry practices followed established regulations: the UK Home Office’s Animals (Scientific Procedures) Act 1986 for experiments performed at the University of Manchester; and the Protection of Animals Used for Scientific or Educational Purposes Act (15 January 2015) and the Polish Local Ethics Committee for Experimentation on Animals no. 1 (Warsaw, Poland) for experiments performed at the University of Warsaw. Moreover, this study was approved by both committees (Permission Number 545/2014 in Poland and licence number 40/3409 in the UK).

### Preparation of dsRNA and mRNA

Double-stranded RNA (dsRNA) against *E-cadherin* and *mCherry* transcripts were synthesised using a MEGAscript RNAi Kit (Ambion) following the manufacturer’s protocol. DNA templates (~500 kb) were produced by PCR, and the core T7 promoter sequence was added at the 5’ ends. The template was amplified from either pCS2EcadHA or pCS2-Cherry vectors using the following primers:

dsE-cadherin

5’-TAATACGACTCACTATAGGGAGACTGCTGCTCCTACTGTTTCT-3’ & 5’-TAATACGACTCACTATAGGGAGAGAACACCAACAGAGAGTCGT-3’ and

dsCherry

5’-TAATACGACTCACTATAGGGAGACCGACTACTTGAAGCTGTCCTT-3’ & 5’-TAATACGACTCACTATAGGGAGAGTTCCACGATGGTGTAGTCCTC-3’

mRNA for H2B-GFP was used as the clonal cell tracker and was *in vitro* transcribed from pCS2H2B-GFP (SP6 promoter) using the mMESSAGE mMACHINE Kit (Ambion). For microinjections, all nucleic acids were diluted in nuclease-free water at final concentrations of: 340 ng/μl (*dsE-cadherin*), 500 ng/μl (*dsCherry*) and 500 ng/μl (*H2B-GFP* mRNA).

### Microinjections

A dsRNA microinjection of a single 8-cell embryo blastomere, leading to specific clonal interference with gene function, was performed as described previously [34, 35, 47] using the FemtoJet injector (Eppendorf), under an inverted Leica DMI6000B or Zeiss Axiovert 200 microscope equipped with Leica or Eppendorf micromanipulators, respectively. Microinjections were carried out in micro-drops of M2 medium, after which the embryos were cultured as described above.

### Immunostaining

Embryo *zonae pellucidae* were removed using acidic Tyrode’s solution, as previously described [48], and tissue fixation was performed in 4% PFA for 30 minutes at room temperature. The specific protein antibodies were as follows: for E-cadherin (Zymed, 18–0223) [27], OCT4 (Santa Cruz Biotechnology, sc-5279) [27, 49, 50], GATA4 (Santa Cruz Biotechnology, sc-1237) [49,50], SOX2 (Abcam, 97959) [51], SOX17 (R&D Systems, NL1924R) [52], Twist (Abcam, ab50581) [53], N-cadherin (BD Transduction Laboratories, 610920) [54,55], Snail (Cell Signaling, 4719) [56] and Vimentin (Cell Signaling, 5741) [57]. Briefly, embryos were permeabilised in 0.55% Triton X-100 (Sigma) solution in PBS for 20 minutes, quenching unreacted aldehydes in NH_4_Cl for 10 minutes, and blocked in 10% foetal bovine serum (FBS, Gibco) for 40 minutes. Primary antibodies were used at a concentration of 1:100, except for SOX17 (1:200) and E-cadherin (1:500). Embryos were incubated with primary antibodies overnight at 4°C. Alexa Fluor-conjugated secondary antibodies (Invitrogen: donkey anti-goat Alexa 488, donkey anti-rabbit Alexa 568, donkey anti-mouse Alexa 568, donkey anti-rabbit Alexa 647, donkey anti-rat Alexa 633, Jackson ImmunoResesarch Laboratories: goat anti-mouse TRITC, goat anti-mouse FITC) were used in dilutions of 1:500. DNA was visualised using Hoechst 33342 (10 μM, Molecular Probes) or DRAQ5 (20 μM, BioStatus).

For three-dimensional (3D) imaging, an A1R Nikon inverted confocal microscope was used. Embryos were imaged in their entirety with a 20x objective using Z-stacks of 3 μm thickness.

### Measurement of the relative level of E-cadherin

**Fig 1:**
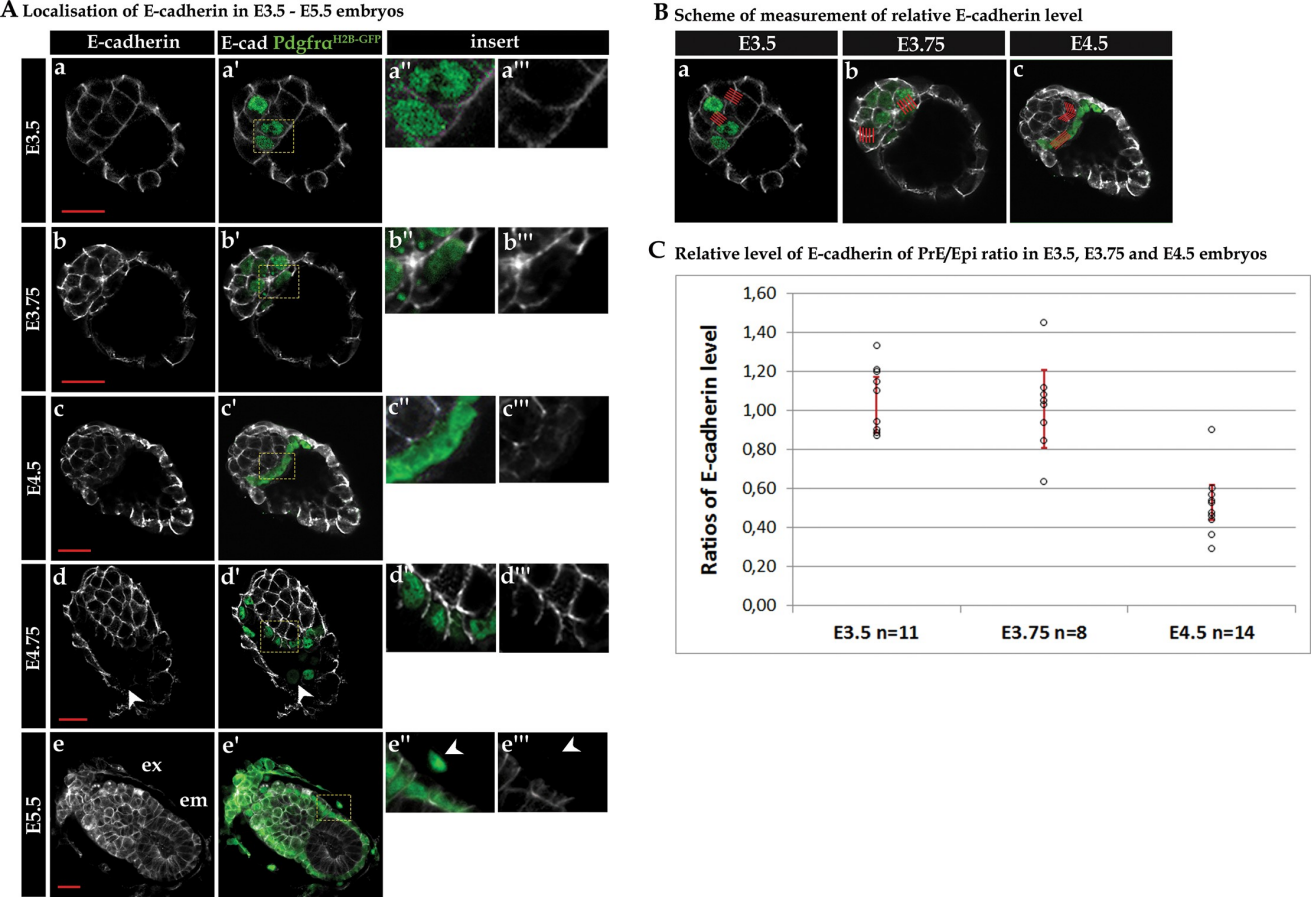
E-cadherin localisation and distribution in E3.5–E5.5 embryos. (A) Immunofluorescence for E-cadherin in embryos at E3.5 (n = 5, a–a”‘), E3.75 (n = 9, b–b”‘), E4.5 (n = 10, c–c”‘), E4.75 (n = 7, d–d”‘) and E5.5 (n = 8, e–e”‘). PrE cells/precursors are GFP-positive (Pdgfrα^**H2B-GFP**^). The arrow denotes a parietal endoderm cell lacking E-cadherin. Ex–extraembryonic part, Em–embryonic part. (B) Scheme for measuring fluorescent signal to determine the relative levels of E-cadherin in E3.5, E3.75 and E4.5 embryos. Red lines indicate measurement of fluorescent intensity between two Epi and two PrE cells. (C) Estimated levels of E-cadherin in PrE cells relative to Epi cells in E3.5, E3.75 and E4.5 embryos; 95% confidence intervals are plotted for the ratios.

Due to attenuation (*i*.*e*., the gradual loss of fluorescent signal through the Z-plane of the embryo), all three lineages—Epi, PrE and TE—were measured using the same Z-slice. Accordingly, we chose slices on which cells from all three lineages (Epi, PrE and TE) were clearly visible. For E3.5 and E3.75 embryos, measurements were only possible for one pair of cells per lineage, and for E4.5 embryos, for two pairs (Fig 1B, a, b, c). Quantifications were executed at five designated points of contact in the regions comprising two neighbouring Epi cells, two PrE cells or two TE cells (Fig 1B, a and b). When measuring across the contact region of two cells, five equidistant lines were drawn and the level of fluorescent signal was measured; the maximum intensity peak for each line was selected for quantification. To compare the fluorescence intensities between two cells of different lineages (PrE, Epi and TE) and between embryos at different stages of development (E3.5, E3.75 and E4.5), we calculated interval estimates (95% confidence interval) for the respective ratios of geometric means, using the Satterthwaite approximation to avoid dependence on an assumption of homoscedasticity [58].

For the experiments in which *E-cadherin* expression was downregulated using dsRNA, the relative level of membrane E-cadherin in injected embryos was measured on Z-slices visualised in at least two daughter cells and in both injected and non-injected blastomeres. The fluorescent signal intensity was measured at five points of contact (as described above) between two precursor cells of the initially microinjected blastomere and two precursor cells of the non-microinjected blastomere. The level of E-cadherin in treated *vs* untreated blastomeres was estimated with confidence intervals for the ratio of geometric means, as above.

When comparing features of subsequent stages of embryo development, changes were expressed as odds ratios for cells belonging to particular groups. These are presented “backwards”, *i*.*e*., as a ratio of the earlier stage divided by the ratio of a later stage. Confidence intervals for these ratios were found using the SAS/FREQ procedure, according to the exact method which was previously described [59].

### Contribution of blastomeres injected with dsRNA

Embryos injected with *dsEcad* or *dsCherry* were fixed at the end of the culture, immunostained for GATA4 and OCT4 proteins, and imaged using the A1R Nikon confocal microscope. Optical sections were taken every 3 μm. The numbers of cells in the clone of the injected blastomeres that contributed to PrE, Epi and TE were calculated based on GATA4 and OCT4 staining.

### RT-qPCR

Single blastomeres were isolated from E3.5 and E4.5 embryos by immunosurgery and disaggregation. First, the *zonae pellucidae* were removed using acidic Tyrode’s solution [48]. For immunosurgery, blastocysts were incubated in anti-mouse serum followed by incubation in guinea pig complement (both 1:3, 30 minutes, Sigma-Aldrich) [60]. Lysed TE cells were removed by pipetting, and the remaining ICMs were disaggregated by 5-minute incubation in a 0.5% solution of pronase (Calbiochem), transferred to Ca^2+^- and Mg^2+^-free M2 medium supplemented with EGTA (0.2 mg/ml; Sigma-Aldrich) for 15 minutes, followed by disaggregation by repeated pipetting [61]. To isolate a single blastomere after dsRNA injection, only the disaggregation step was performed.

All reagents for RT-qPCR were purchased from Life Technologies, Inc. RNA from individual disaggregated blastomeres was isolated using the Dynabeads (2.8 μm) mRNA DIRECT Micro Kit, following the manufacturer’s protocols. The RT-qPCR protocol employed was described previously [62, 63]. Briefly, kit-eluted mRNA (10 μl H_2_O_depc_) was incubated for 10 minutes with 0.5 μg of oligo(dT)_12-18_ at 70°C. Next, the mixture was supplemented with 1x buffer RT, 10 mM of DTT, 0.5 mM of dNTP mix, 40 IU of RNase inhibitor and 200 IU of the SuperScript II reverse transcriptase to a final volume of 20 μl and incubated at 42°C for 50 minutes. The resulting cDNA was pre-amplified using TaqMan PreAmp Master Mix and pooled TaqMan gene expression assays (Mm01205647 for β-actin, Mm99999915 for GAPDH, Mm00802636 for *Gata6*, Mm 00484689 for *Gata4*, Mm01247357 for *E-cadherin*, Mm001162497 for *N-cadherin*, Mm01249009 for *P-cadherin*, Mm00486932 for *R-cadherin*, Mm00486938 for *VE-cadherin*, Mm00515466 for *OB-cadherin* and Mm00490584 for *T-cadherin*, each at the x0.2 concentration) for 18 cycles: 95°C for 15 seconds, 60°C for 4 minutes. For qPCR, the TaqMan Gene Expression Master Mix in the OneStep Real-Time PCR System (Applied Biosystems) was used. Reactions were set in 10 μl volume, and the amplification was run for 50 cycles: 95°C for 15 seconds, 60°C for 1 minute. The relative level of expression was measured using the 2^-ΔCt^ method [64]. For normalization, we used the geometric mean of the relative levels of β-actin and GAPDH expression [65].

## Results

### E-cadherin is distributed uniformly in the cell membrane of Epi and PrE precursors at the early blastocyst stage

E-cadherin is the major protein responsible for adhesion in mammalian cells [31]. A targeted mutation in the mouse *E-cadherin* gene results in defective preimplantation development [27, 34, 66]. However, thus far, the impact of E-cadherin loss has only been investigated in relation to the specification of the ICM *vs* TE and not during PrE *vs* Epi specification. To assess the role of E-cadherin during ICM cell lineage specification, we first investigated its localisation at consecutive stages of preimplantation and peri-implantation embryos using immunocytochemistry. To identify the PrE population, we used embryos derived from the transgenic Pdgfrα^H2B-GFP/+^ strain [42], which express GFP under the control of the promoter of platelet-derived growth factor α receptor (*Pdgfrα*), an early marker of PrE fate [6]. At the early blastocyst stage (E3.5, 30–63 cells), PrE marker expression was observed in some ICM cells, and in accordance with our previous results, we could distinguish three populations: GFP-ve cells, classified as Epi precursors; GFP+ve low cells, which may contribute to both Epi and PrE lineages; and GFP+ve high cells, classified as PrE precursors [6, 51]. At this early blastocyst stage, E-cadherin was uniformly distributed along the cell membrane irrespective of the GFP expression level, indicative of both PrE and Epi precursor cell populations (Fig 1A, a-a”‘). None of the ICM cells exhibited a polarised distribution of E-cadherin based on their relative position (*i*.*e*., either deep within the ICM or on the surface layer facing the cavity). Conversely, in TE cells, E-cadherin was localised only at the basolateral part of the cell membrane (Fig 1A, a-a’). Taken together, our observations indicate that at the early blastocyst stage, PrE and Epi cells do not differ in membrane E-cadherin distribution and do not polarise when lining the blastocyst cavity.

### E-cadherin distribution becomes polarised at the mid-blastocyst stage after PrE cells reach their final surface position within the ICM

At the mid-blastocyst stage (E3.75, 64–100 cells) the PrE and Epi precursors were distributed within the ICM in a seemingly random “salt-and-pepper” manner, in agreement with previously published data [7]. In Epi cells, localised both deep in the ICM and on its surface, E-cadherin was evenly distributed in the membrane, just as it was in Pdgfrα-negative Epi cells at the earlier stage (Fig 1A, b-b”‘). In PrE cells, we distinguished two patterns of E-cadherin localisation. In cells localised inside the ICM, E-cadherin was evenly distributed, similar to Epi cells. However, in Pdgfrα-positive PrE cells localised on the surface of the ICM, a polarised distribution of E-cadherin was observed: it was present in the basolateral but not in the apical part of the membrane (Fig 1A, b-b”‘).

At the late blastocyst stage (E4.5, 101–140 cells), PrE and Epi cells were clearly segregated within the ICM as separate, well-defined tissue layers, with the PrE cells present as an epithelium lining the surface of the ICM facing the blastocyst cavity and separating the Epi population from the cavity, as previously described [35, 67, 68]. At this stage, E-cadherin was distributed evenly in the membrane of Epi cells and was polarised in PrE cells. Moreover, we noted that the intensity of the fluorescent signal of E-cadherin immunostaining was much stronger in Epi cells than in PrE cells (Fig 1A, c-c”‘), indicating a reduction in PrE cell membrane-associated E-cadherin protein expression/localisation.

To summarise, we concluded that E-cadherin distribution was visibly different between precursors and mature PrE and Epi cells once the process of lineage segregation had been initiated. Namely, Epi cells contained membrane E-cadherin that was evenly distributed, whereas cells within the PrE layer only displayed E-cadherin protein in the basolateral, but not the apical, parts of the membrane (Fig 1A, c-c”‘), which is consistent with their epithelial character.

### The relative levels of E-cadherin in cell-cell contact in PrE to Epi lineages change during PrE formation

In early blastocysts, we observed an even distribution of E-cadherin in both PrE and Epi precursors and the protein level seemed to be similar, suggesting such precursors are unlikely to differ in their cell adhesion properties. However, at later stages, when cells within the ICM are segregated, we observed differences in E-cadherin localisation between Epi and PrE cells. It is therefore possible that, contrary to previous suggestions [6–8, 39–41], Epi and PrE cells segregation does not require relative differences in cell adhesion mediated by heterogeneous levels of E-cadherin. To gain further insight about the levels of E-cadherin in both PrE and Epi cell populations, we measured the relative levels of E-cadherin localised at regions of cell-cell contact between Epi and PrE cells before and after ICM cell lineage segregation.

We measured the relative levels of E-cadherin between TE, Epi and PrE cells (Fig 1B) and computed 95% confidence intervals for PrE/Epi, Epi/TE and PrE/TE ratios (see Materials and Methods). Next, we compared the relative levels of these ratios in the subsequent stages of blastocyst development. In Fig 1C, we present data for the PrE/Epi ratio, whilst all remaining data for PrE/Epi, Epi/TE and PrE/TE ratios can be found in the supplementary material (S1 Fig). A PrE/Epi mean ratio close to 1 indicates that the level of E-cadherin between Epi and between PrE cells is similar, whereas a mean ratio of less than 1 is indicative of Epi cells having a greater level of E-cadherin than PrE cells. Conversely, a mean ratio of more than 1 means a higher level of E-cadherin in PrE cells than in Epi cells.

Accordingly, we observed that at the E3.5 blastocyst stage, the mean PrE/Epi ratio was not much different from 1, meaning that the relative level of E-cadherin was similar in PrE and Epi cells (Fig 1C). At E3.75, the inter-embryo variation was greater, exhibiting a range from 0.63 (lower confidence limit) to 1.45 (upper confidence limit), although the calculated mean ratio was still not much different from 1 (Fig 1C). However, in E4.5 stage blastocysts, the PrE/Epi ratio was decidedly lower (with an upper confidence limit of 0.9; Fig 1C), illustrating lower levels of E-cadherin in PrE cells than in Epi cells. We next compared the relative levels of E-cadherin localised in cell-cell contact regions for PrE/Epi ratios according to the stages of embryo development (E3.5, E3.75 and E4.5). We computed 95% confidence intervals for the respective ratios of the geometric means. Using this method, it was not possible to unequivocally exclude the change in the PrE/Epi ratio between stages E3.5 and E3.75 (confidence interval for their ratios: 0.88, 1.21). However, there was a substantial decrease in the relative levels of E-cadherin in Epi and PrE cell-cell contact regions between stages E3.5 and E4.5 and also between stages E3.75 and E4.5 (lower confidence limits: 1.46 and 1.7, respectively). Thus, our results indicate that before segregation of the PrE and Epi precursors, the levels of E-cadherin at cell-cell contact regions do not differ considerably between these two precursor lineages. Moreover, a clear difference could only be observed after the PrE and Epi cells segregation. Lastly, we found that when PrE and Epi cells were segregated, the level of E-cadherin decreased between PrE cells in comparison with Epi cells, although it is not possible to conclude whether this change is due to the diminution in PrE cells *vs* Epi cells, or whether Epi cells retain higher levels of membrane E-cadherin.

### Downregulation of E-cadherin does not influence cell segregation and is not sufficient to trigger PrE specification

**Fig 2:**
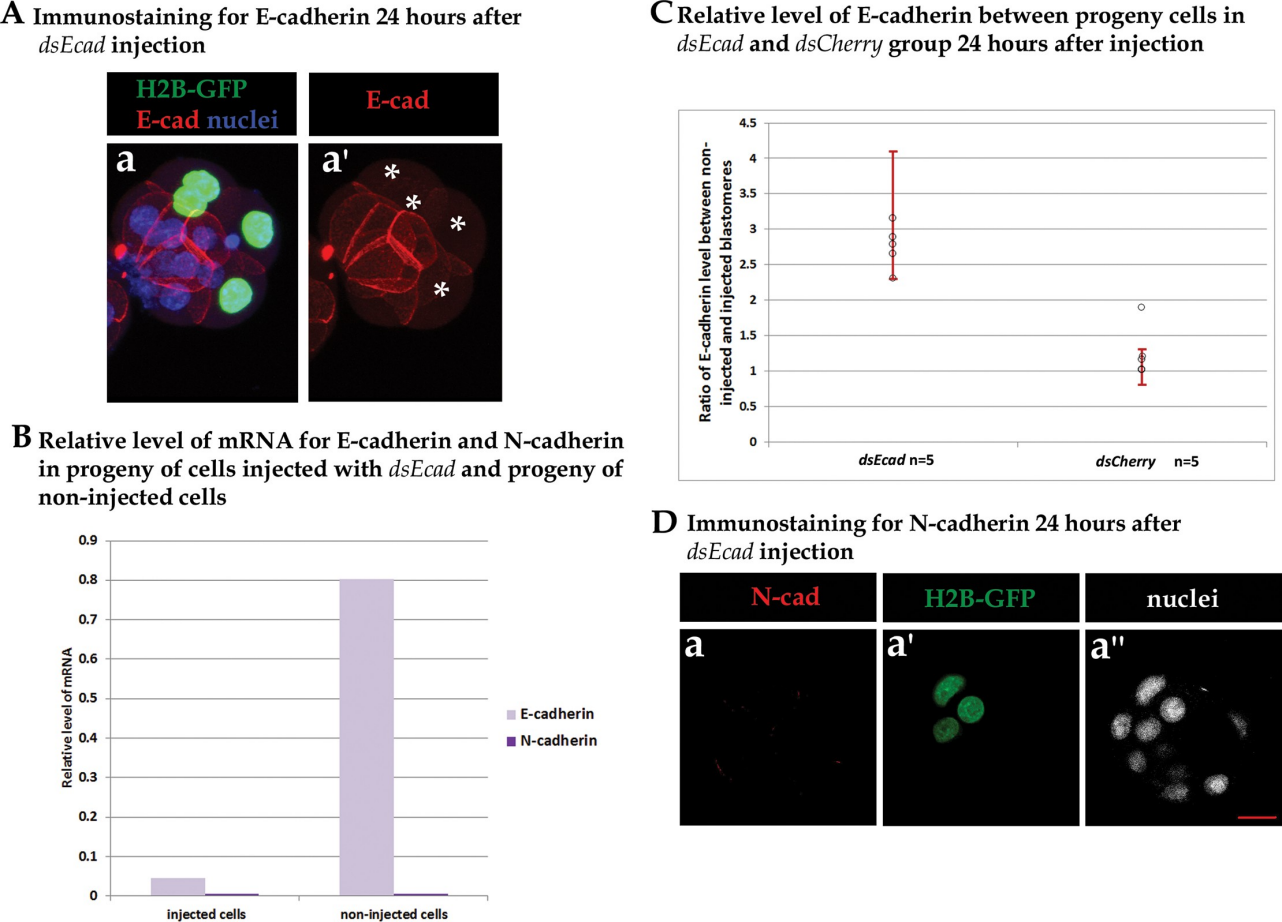
Downregulation of *E-cadherin* by microinjection of dsRNA. (A) Immunostaining for E-cadherin 24 hours after *dsEcad* injection. Stars are used to mark the progeny of injected blastomeres. (B) mRNA levels for *E-cadherin* and *N-cadherin* in the progeny of cells injected with *dsEcad* and in the progeny of non-injected cells from the same embryos. C) Plotted are 95% confidence intervals for the ratio of E-cadherin level between non-injected and injected cells. (D) Immunostaining for N-cadherin in embryos injected with *dsEcad*.

E-cadherin localisation is altered in PrE cells that are in contact with the cavity. Since it has been previously proposed that cell adhesion contributes to cell segregation, we asked whether downregulation of E-cadherin might be a trigger for the segregation of Epi and PrE during the final stages of PrE cells differentiation. *E-cadherin* can be efficiently downregulated using RNAi techniques [47]. Thus, we performed RNAi-mediated downregulation of *E-cadherin* expression to study its effect on the specification and segregation of cells within the ICM. We purposefully chose downregulation rather than complete knockout, as a total lack of *E-cadherin* is known to cause the decompaction of blastomeres in mouse embryos [27, 28, 34]. To this end, we microinjected one randomly chosen blastomere at the 8-cell stage with dsRNA (*dsEcad* group) together with mRNA for the histone H2B tagged to GFP (H2B-GFP; Fig 2A [34]. We decided to initiate downregulation at the 8-cell stage, and not earlier, to maximise the probability that functional downregulation of *E-cadherin* would be achieved at the point in development when ICM cells are spatially segregating. We then traced the progeny of this cell, using H2B-GFP as a live marker, to determine whether it contributed to either the PrE or the Epi lineage at the late blastocyst stage. As a control, we microinjected dsRNA designed against the fluorescent protein mCherry (*dsCherry* group) transcript using the same lineage tracer mRNA.

To confirm that the *dsEcad* RNA used was ee ffective in reducing *E-cadherin* transcripts, we measured *E-cadherin* mRNA levels by RT-qPCR in the progeny of injected blastomeres 24 hours after injection. We confirmed that our RNAi treatment resulted in a 95% (17-fold) decrease in the relative abundance of mRNA for *E-cadherin* in treated cells compared to untreated control blastomeres (Fig 2B).

Consistently, the microinjection of *dsEcad* RNA also led to significantly lower levels of E-cadherin protein after 24 hours of *in vitro* culture, as assessed by immunocytochemistry (Fig 2A). By this developmental stage, the treated blastomeres had already undergone one or two rounds of cell division, and analysis of the relative levels of E-cadherin protein in the progeny of the treated blastomeres revealed downregulated expression: the protein levels in the treated blastomeres were 1.73 to 3.4-fold lower than those of the control blastomeres (Fig 2C). We next analysed whether downregulation of *E-cadherin* is maintained in ICM cells (marked by SOX2) after prolonged culture, before their final segregation to either the Epi or PrE (S2 Fig). We analysed the cell-cell contact regions between two progenies of non-microinjected cells and between two progenies of microinjected cells, as well as between a mix of microinjected and non-microinjected cells. This analysis revealed that *E-cadherin* downregulation is maintained in the embryo at least until the early blastocyst stage: the low end of the confidence interval was more than 1, meaning there was a higher level of E-cadherin between the two non-microinjected cells compared to the level of E-cadherin between the two microinjected cells (S2B Fig). Similarly, the low limit of the confidence interval was more than 1 when non-injected and mix cells were analysed.

To examine the possible effect of *E-cadherin* downregulation on cell fate specification within the ICM, we cultured RNAi-treated embryos for 52–54 hours (until the late blastocyst stage) and examined how the progeny of the treated cells contributed to the three blastocyst lineages, assaying the expression of lineage-specific markers for PrE and Epi (*i*.*e*., GATA4 and OCT4, respectively). Blastomeres expressing *Gata4*, forming a monolayer on the surface of the ICM, were classified as PrE cells, whereas blastomeres inside the ICM, expressing *Oct4*, were categorised as Epi cells. Cells forming the outside monolayer of the embryo that were negative for GATA4 and OCT4 were classified as TE cells. We analysed the contribution of blastomeres with lower level of E-cadherin to each lineage (Epi, PrE and TE). The odds of being a descendant of an injected blastomere found within a lineage was a starting point; we treated the respective odds ratios as characteristics of cells’ “preferences” within the particular lineages. We found evidence that the *dsEcad* microinjected blastomere progeny contributed slightly more to the TE lineage that to other lineages (Table 1).

**Table 1:** Lineage allocation of blastomeres after E-cadherin downregulation.

	Sample OR	LCL OR	UCL OR	p-value
Epi	0.82	0.41	1.7	0.61
PrE	1.35	0.74	2.56	0.33
TE	1.33	1.01	1.76	0.04

The odds ratios (ORs) presented are the odds of being a descendant of an injected blastomere within the dsEcad clone divided by the respective odds in the dsCherry clone. LCL and UCL denote, respectively, lower and upper 95% confidence limit computed by the exact method. The total numbers of cells (dsEcad, dsCherry) were: (391, 169) for Epi; (309, 138) for PrE and (2,206, 1,307) for TE.

Based on the criteria described above, we also analysed whether all progeny of the injected cells were positioned appropriately within the ICM, *i*.*e*., blastomeres expressing PrE markers were on the surface of the ICM and blastomeres expressing Epi markers were deep within the ICM. We did not observe any incidence of mispositioned cells caused by *E-cadherin* downregulation. Thus overall, we concluded that downregulation of *E-cadherin* during the preimplantation period does result in a slightly higher contribution of descendant microinjected cells to the TE lineage, but that it does not cause any bias in the allocation between the Epi and PrE.

### N-cadherin does not compensate for E-cadherin downregulation

As a compensatory mechanism has been described previously [69], by which increased *N-cadherin* expression compensates for depleted E-cadherin, we examined whether *N-cadherin* is upregulated in response to RNAi-mediated *E-cadherin* downregulation. However, we did not detect the presence of N-cadherin protein, as assessed by immunofluorescence, 24 hours after *dsEcad* microinjection in the progeny of either injected or non-injected blastomeres (Fig 2D; a positive immunostaining control for the anti-N-cadherin antibody is presented in S3 Fig). Furthermore, RT-qPCR analysis also failed to show any detectable *N-cadherin* mRNA in *dsEcad* microinjected and non-microinjected control cells (Fig 2B). These data clearly indicate that in our experimental model, the downregulation of *E-cadherin* is not accompanied by an upregulation of *N-cadherin* expression and consequently negates the possibility of a compensatory role of this protein in this developmental context.

### E-cadherin is present in the VE but not in PE cells of peri-implantation stage embryos

Given that we had demonstrated that formation of an epithelium by PrE cells lining the cavity is accompanied by intracellular E-cadherin rearrangement, we next asked whether E-cadherin is involved in the formation of descendent VE and PE cells. Indeed, we observed that by the E4.75, a subset of PrE cells that had migrated away from the ICM to form the PE had clearly diminished or undetectable levels of E-cadherin protein (Fig 1A, d-d”‘). Conversely, the PrE cells that had remained on the surface of the ICM and had formed the VE displayed a polarised pattern of E-cadherin distribution, with lower level of E-cadherin, similarly to what we observed in PrE cells lining the ICM earlier, at the late blastocyst stage. Namely, E-cadherin was present in the basolateral regions and absent from the apical part of the plasma membrane: a pattern of expression that persisted to the E5.5 stage in the emergent VE cell population. We did not observe any detectable E-cadherin signal in either the basolateral or apical membranes of PE cells—cells which by this stage (E5.5) are involved in the Reichert’s membrane development (Fig 1A, e-e”‘).

### The PrE-to-PE transition resembles epithelial-to-mesenchymal transition (EMT)

**Fig 3:**
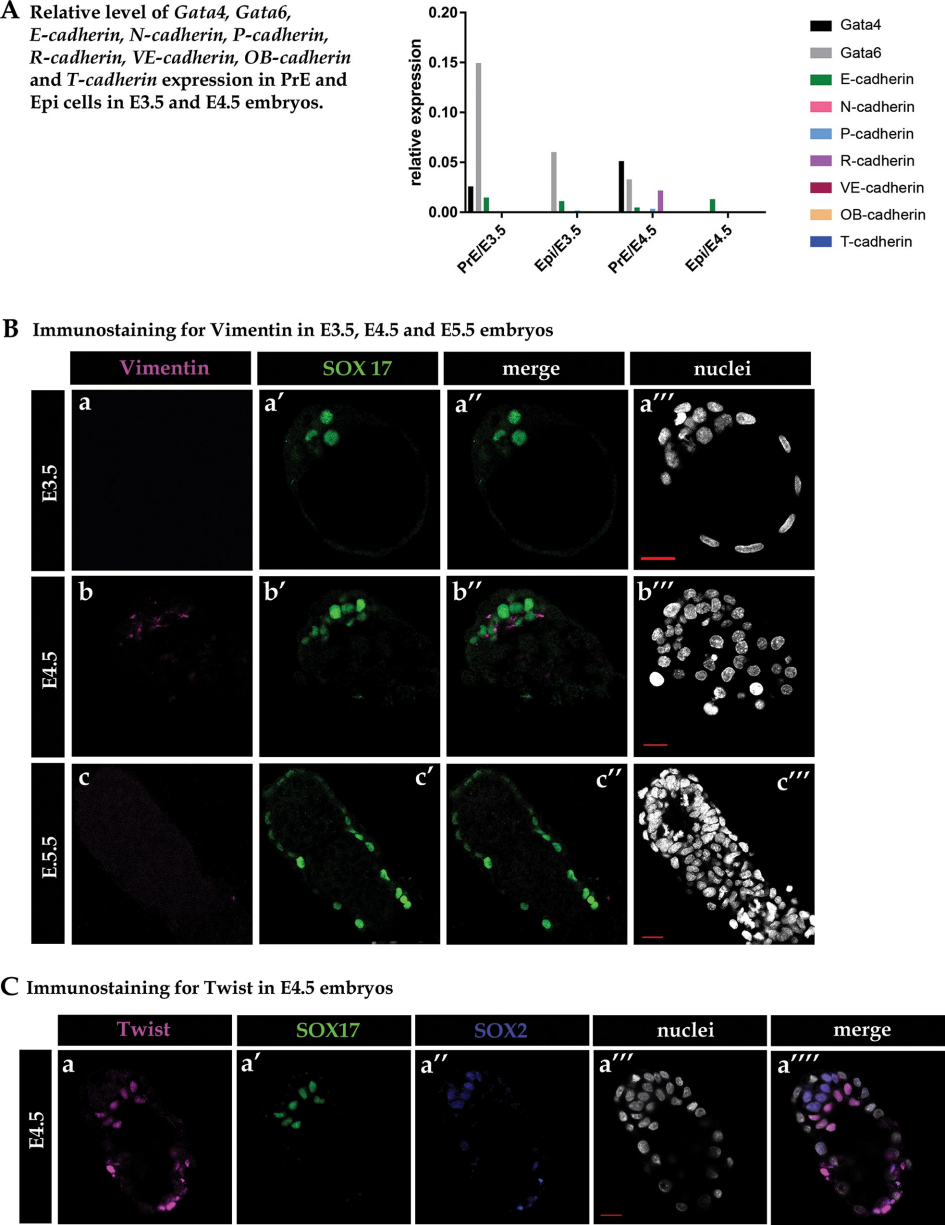
Relative levels of *cadherin* gene expression and immunostaining of EMT proteins. (A) Relative levels of mRNA for *Gata4*, *Gata6*, *E-cadherin*, *N-cadherin*, *P-cadherin*, *R-cadherin*, *VE-cadherin*, *OB-cadherin* and *T-cadherin* in Epi and PrE populations of E3.5 and E4.5 embryos. (B) Immunostaining for Vimentin in E3.5, E4.5 and E5.5 embryos. (C) Immunostaining for Twist (a–a”“) in the E4.5 blastocyst.

**Fig 4:**
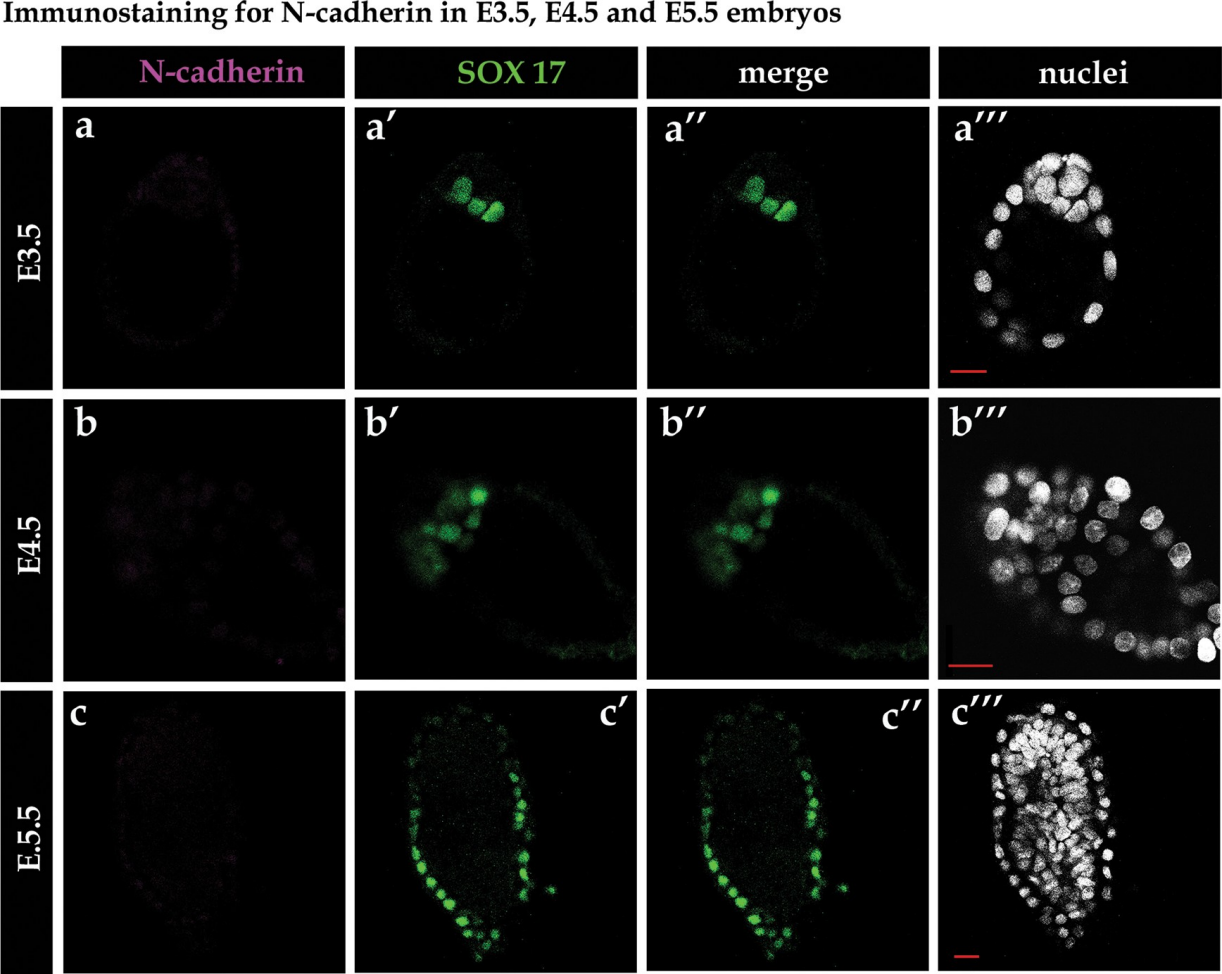
Immunostaining for N-cadherin in mouse embryos. Immunostaining shows a lack of N-cadherin in E3.5, E4.5 and E5.5 mouse embryos.

As we had observed changes in E-cadherin distribution between Epi and PrE cells and then later, between VE and PE cells, and because re-localisation and downregulation of E-cadherin are hallmarks of EMT, we decided to investigate whether the PrE-to-PE transition also resembles EMT. First, we checked if the cadherin switch, known to occur in the paradigms of EMT, also occurs in the PrE cells of E4.5 stage embryos. To this end, we applied qPCR to verify the presence of transcripts of other cadherins in PrE cells. According to previous single cell-derived data, *N-cadherin*, *P-cadherin*, *R-cadherin*, *VE-cadherin*, *OB-cadherin* and *T-cadherin* transcripts are detectable in PrE cells at the late blastocyst stage, but each transcript is detected in a different individual cell [39]. For this reason, we decided to collect populations (six blastomeres) of Epi or PrE cells from E4.5 stage blastocysts. To ascertain which cadherins potentially become newly expressed in the PrE lineage, the results were compared with the equivalent data obtained for blastomeres collected at E3.5. To collect PrE and Epi cells from E3.5 and E4.5 stage blastocysts, we removed TE cells by immunosurgery and then disaggregated isolated ICMs to collect single cells. We used transgenic Pdgfrα^H2B-GFP/+^ embryos [42], described above, to identify PrE cells (GFP+ve) [6]. Blastomeres negative for GFP signal (GFP-ve) were classified as Epi cells. Out of six cadherins assayed (*N-cadherin*, *P-cadherin*, *R-cadherin*, *VE-cadherin*, *OB-cadherin* and *T-cadherin*), we detected only *R-cadherin* and very low levels of *P-cadherin* transcripts in the PrE population of the E4.5 stage blastocyst (Fig 3A). We did not detect any *N-cadherin* expression at the mRNA or protein level (Figs 3A and 4).

We subsequently verified whether other EMT markers, *i*.*e*., Vimentin, Twist and Snail, were present in cells undergoing the PrE-to-PE transition. We performed co-immunostaining of these markers, together with SOX17 (a PrE marker). We discovered that Vimentin protein was not present in E3.5 (n = 7) or E5.5 (n = 6) stage embryos (Fig 3B, a-a”‘ and c-c”‘), but it was detectable in the PrE cells of E4.5 stage embryos in 8 out of the 19 blastocysts analysed (Fig 3B, b-b”‘), as was the presence of Twist in all PrE cells in every analysed E4.5 embryo (n = 7, Fig 3C, a-a”“). However, we did not observe the presence of Snail at this stage, at least with the antibody we have used (S3B Fig).

In summary, these data support the hypothesis that an EMT process does indeed occur in the peri-implantation stage mouse embryo. However, more investigation is required to further characterise events during the PrE-to-PE transition.

## Discussion

E-cadherin is the main protein responsible for the adhesion properties of the mammalian embryo. Here, we investigated a possible role of E-cadherin in the specification and segregation of PrE *vs* Epi cells, and subsequently the specification of VE *vs* PE, during the peri-implantation stages of mouse embryo development. At the early and mid-blastocyst stage, when Epi and PrE precursors are distributed within the ICM in a “salt-and-pepper” manner, we observed no considerable differences in E-cadherin protein levels in the cell-cell contact regions between cells of these two lineages. We observed a marked decrease in the relative levels of E-cadherin in PrE cells when compared with Epi cells, but only after their segregation towards a cavity-proximal position within the ICM. Additionally, the distribution of ICM cell membrane-associated E-cadherin was shown to change: during segregation, the previously uniform pattern gave way to a typically polarised distribution in PrE cells facing the cavity. However, since the changes in E-cadherin level only occur after PrE cells’ repositioning at the ICM surface, a significant role for inter-cell E-cadherin heterogeneities—and hence the relative differences in cell adhesion in mediating Epi/PrE segregation—seems improbable.

It has been previously reported that the aggregation of cells exhibiting different adhesive properties results in an aggregate characterised by a superficial layer of less adherent cells, whereas a population of highly adhesive cells localises within the aggregate [70]. Indeed, it has been suggested that similar mechanisms may be employed during Epi and PrE cell segregation [6, 41, 71]. To test this assumption, we artificially decreased the adhesion properties of cells by creating a clone of cells in which *E-cadherin* had been successfully downregulated. We aimed to assess whether such experimentally induced changes in E-cadherin levels, within the context of a normally developing mouse blastocyst, could be sufficient to induce PrE cells differentiation and/or cause interference with the physical segregation of PrE and Epi cells.

Our data revealed a modest bias in the contribution of *E-cadherin* downregulated cell clones towards the TE over the ICM. However, within the ICM, we found no evidence that *E-cadherin* had any effect on cell fate specification or cell lineage segregation in either Epi and PrE cells, *i*.*e*., the induced changes in adhesion properties did not bias blastomeres to differentiate into any particular ICM lineage. Thus, inducing differences in E-cadherin levels between blastomeres is not enough to alter their ultimate cell fate specification within the ICM nor is to influence any subsequent mechanisms to sort the cells into the two separate tissue compartments.

It has previously been reported that the upregulation of *N-cadherin* can compensate for a lack of E-cadherin in embryonic stem cells [69]. In this paradigm, N-cadherin adopts the functional role of E-cadherin in supporting pluripotency. In our study, we found no such evidence of compensatory *N-cadherin* expression in response to the downregulation of *E-cadherin* by dsRNA in the mouse blastocyst. However, we cannot exclude the possibility of other potential compensation mechanisms, possibly invoked by other cadherins. As there are no differences in E-cadherin levels between Epi and PrE precursor cells before their segregation, and as downregulation of *E-cadherin* affects neither ICM cell specification nor segregation, we speculate that differential adhesion properties are not required for the segregation of cells within the ICM. Apart from E-cadherin, it is possible that other proteins, *e*.*g*., Ephrin or Integrin-related proteins, may also be involved in ICM cells specification and segregation. However, it has been shown that the level of *Ephrin* mRNA does not differ between Epi and PrE cells in the E4.5 blastocyst [72]. Similarly, Integrin B1 has also been investigated in the context of mouse preimplantation development, but a role of this adhesion protein in the Epi and PrE cells segregation has been excluded [73, 74]. Thus, further investigation of the potential involvement of other Integrin family members during this process may prove fruitful.

We observed that the localisation of PrE cells on the surface of the ICM coincided with the polarised distribution of E-cadherin (characterised by the disappearance of E-cadherin from apical regions of the cell membrane). It is possible that the re-localisation of E-cadherin could be related to a loss of pluripotency in PrE precursors, as they specify a process shown to be at least partially controlled by the WNT pathway [75]. In murine embryonic stem cells, the β-catenin/OCT4 complex is a part of this WNT pathway-mediated regulation [76]. Accordingly, in undifferentiated (pluripotent) embryonic stem cells, the OCT4/β-catenin complex remains associated with membrane E-cadherin [75], only becoming located throughout the cytoplasm in differentiating cells [75]. Thus, the observed polarisation of E-cadherin in the basolateral membranes may reflect the above-described mechanism. It is also possible that clearance of E-cadherin from the apical membrane could be a clathrin-dependent endocytic event [77], mediated by proteolysis [78] or lysosome activity [79]. Indeed, the presence of DAB2 in PrE cells [68] supports the potential involvement of clathrin-dependent endocytosis [67, 80]. However, further investigation of the underlying the mechanisms involved in E-cadherin’s removal from the apical membrane of PrE cells is still needed, with particular emphasis to be placed on its potential role in regulating cell pluripotency, differentiation and selective apoptosis during cell segregation within ICM.

Although E-cadherin does not seem to be required for Epi and PrE cell specification and segregation within the ICM of mouse blastocysts, it may still play a significant role during subsequent PrE cell development; namely the differentiation of the PrE derivatives into the PE and VE. The downregulation of *E-cadherin* and the loss of apical-basal polarity are among the most recognisable events occurring during EMT-related processes. We have shown here that as PrE cells form an epithelial layer on the surface of the ICM, E-cadherin is redistributed and eventually its level decreases in PE cells such that it cannot be detected in E5.5 embryos, confirming that the PrE-to-PE transition is an EMT process.

The intermediate filament protein Vimentin is a known protein marker expressed in cells undergoing EMT [21] and plays a predominant role in inducing cell shape change from epithelial into mesenchymal with motile behaviour. The presence of Vimentin has previously been demonstrated in the gastrulating mouse embryo (E6.0–E7.5) and specifically in cells undergoing EMT [81]; it is additionally expressed during later stages of development (E8.5–E13.5) within the distal endoderm and in primitive streak cells, giving rise to the mesoderm [82, 83]. Indeed, Vimentin has also been shown to be a PE marker between the E9.0 and E.13 stages of mouse development [83, 84]; however, its expression has not been reported at the blastocyst stage [85]. Here, we show that Vimentin is indeed transiently expressed in the mouse blastocyst (at E4.5) and is coincident with the PrE-to-PE transition, a process exhibiting other hallmarks of EMT.

To additionally confirm that EMT occurs during the PrE-to-PE transition, we also verified the presence of Twist, a well-described inducer of EMT that is known to repress *E-cadherin* [86] and upregulate *N-cadherin* and *Fibronectin* [87] expression in other cellular EMT contexts. We found that Twist is present in all PrE cells of E4.5 embryos. Moreover, we uncovered preliminary evidence of a PrE-to-PE transitory ‘cadherin switch’ at this time, typified by the downregulation of *E-cadherin* and the emergence of detectable *R-cadherin* and possibly *P-cadherin* mRNA transcripts. Taken together, these data suggest that EMT mechanisms are potentially invoked during the PrE-to-PE transition.

## Supporting information

S1 Fig Relative levels of E-cadherin in Epi, PrE and TE lineages of the mouse embryo. Estimated levels of E-cadherin in the PrE relative to the Epi in E3.5, E3.75 and E4.5 embryos; 95% confidence intervals are plotted for the PrE/Epi, PrE/TE and Epi/TE ratios.
(JPG)

S2 Fig Effect of downregulation of *E-cadherin* by *dsEcad* after 48 hours of *in vitro* culture. (A) Early blastocyst stained with antibodies against E-cadherin and SOX2 (ICM marker). Red–E-cadherin, green–H2BGFP, blue–SOX2. The same embryo is presented in 2D and 3D mode. (B) 95% confidence intervals of relative levels of E-cadherin in cell-cell contact for ratios: progeny of microinjected blastomere/progeny of non-injected blastomere; a mix of cell-cell contact between microinjected and non-microinjected cells; non-microinjected/mix of cell-cell contact between microinjected and non-microinjected cells.
(JPG)

S3 Fig Immunostaining positive control for the antibody against N-cadherin and immunostaining for Snail in the mouse embryo. (A) HEK cells immunostained with antibody against N-cadherin. (B) Immunostaining for Snail (a–a”“) in the E4.5 blastocyst.
(JPG)
