# Supplementary figures and images for: No evidence of involvement of E-cadherin in cell fate specification or the segregation of Epi and PrE in mouse blastocysts
Source: PLoS One. 2019 Feb 8;14(2):e0212109. doi: 10.1371/journal.pone.0212109 (PMC6368326; doi:10.1371/journal.pone.0212109)

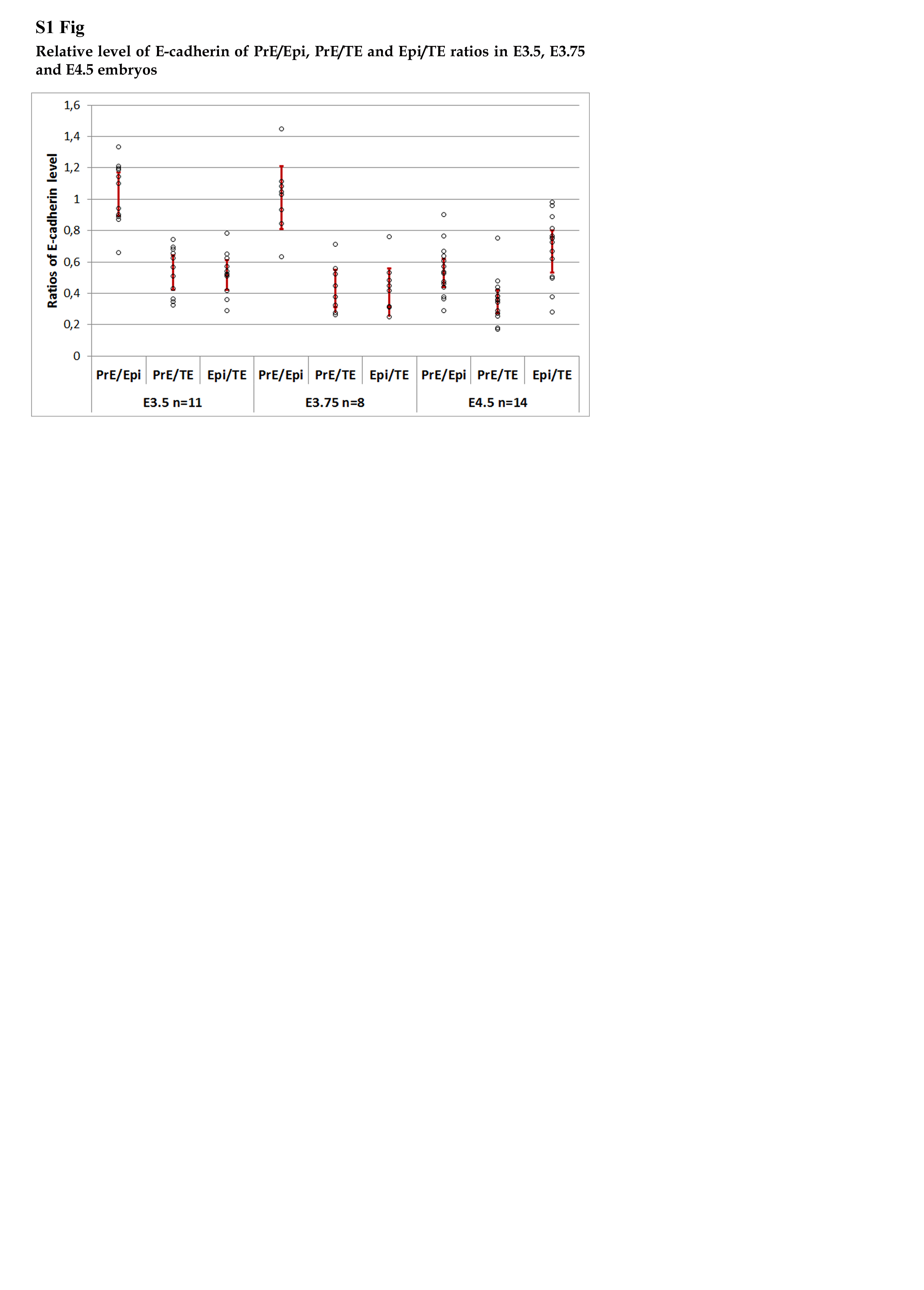

Supplement: S1 Fig — Estimated levels of E-cadherin in the PrE relative to the Epi in E3.5, E3.75 and E4.5 embryos; 95% confidence intervals are plotted for the PrE/Epi, PrE/TE and Epi/TE ratios. (JPG) [file pone.0212109.s001.jpg]

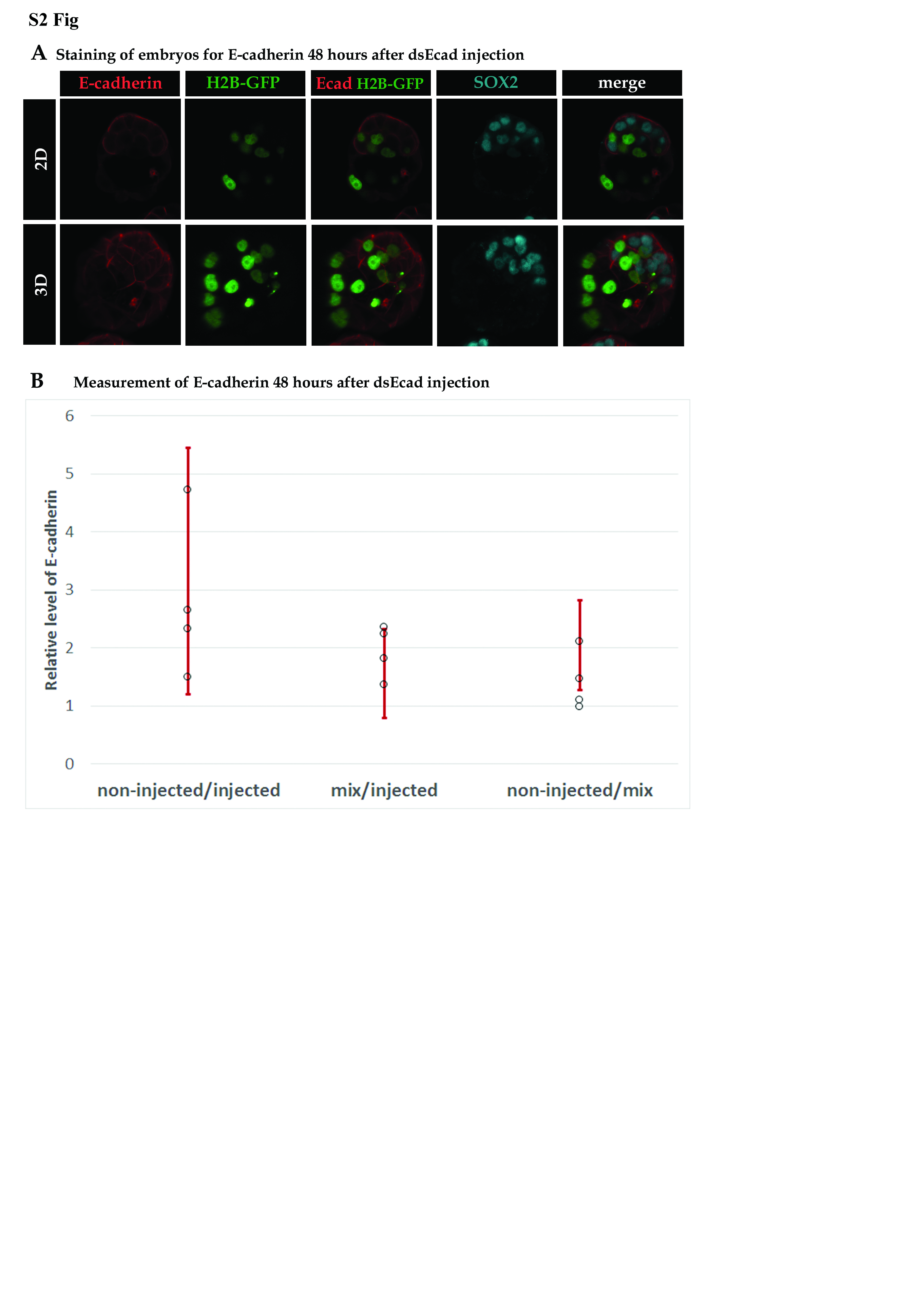

Supplement: S2 Fig — (A) Early blastocyst stained with antibodies against E-cadherin and SOX2 (ICM marker). Red–E-cadherin, green–H2BGFP, blue–SOX2. The same embryo is presented in 2D and 3D mode. (B) 95% confidence intervals of relative levels of E-cadherin in cell-cell contact for ratios: progeny of microinjected blastomere/progeny of non-injected blastomere; a mix of cell-cell contact between microinjected and non-microinjected cells; non-microinjected/mix of cell-cell contact between microinjected and non-microinjected cells. (JPG) [file pone.0212109.s002.jpg]

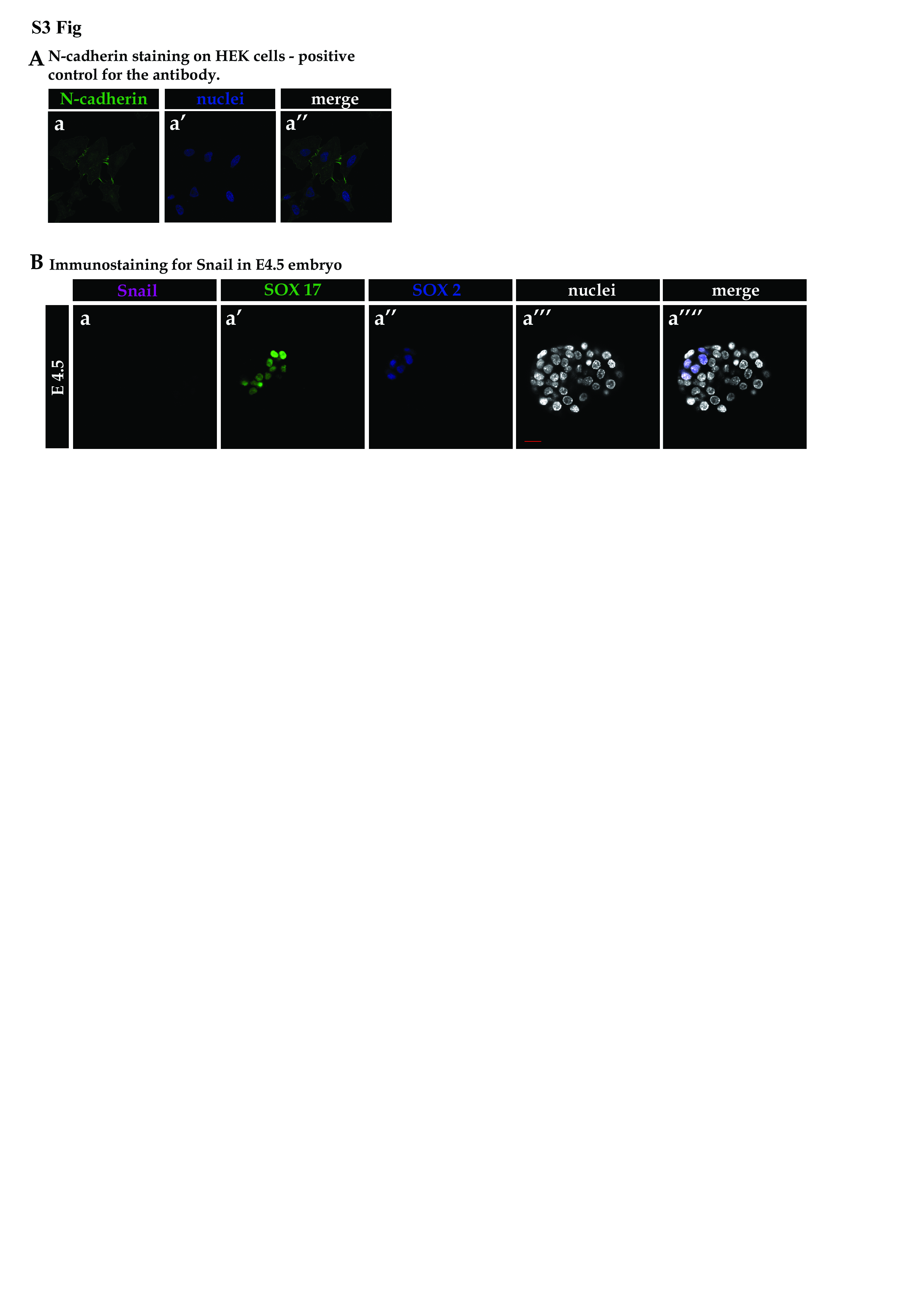

Supplement: S3 Fig — (A) HEK cells immunostained with antibody against N-cadherin. (B) Immunostaining for Snail (a–a”“) in the E4.5 blastocyst. (JPG) [file pone.0212109.s003.jpg]
